# Antibiotic resistance of *Helicobacter pylori* in Nanjing, China: a cross-section study from 2018 to 2023

**DOI:** 10.3389/fcimb.2023.1294379

**Published:** 2023-11-28

**Authors:** Wenjuan Wei, Zhibing Wang, Chao Li, Zongdan Jiang, Zhenyu Zhang, Shukui Wang

**Affiliations:** ^1^ Department of Gastroenterology, Nanjing First Hospital, Nanjing Medical University, Nanjing, Jiangsu, China; ^2^ Department of Laboratory Medicine, Nanjing First Hospital, Nanjing Medical University, Nanjing, Jiangsu, China; ^3^ Jiangsu Collaborative Innovation Center on Cancer Personalized Medicine, Nanjing Medical University, Nanjing, Jiangsu, China

**Keywords:** *Helicobacter pylori*, antibiotics resistance, levofloxacin, PCR, *gyrA* mutations

## Abstract

**Background:**

The increasing prevalence of antibiotic resistance in cases of *Helicobacter pylori* (*H. pylori*) infection has emerged as a significant global issue. This study offers a comprehensive examination of the alterations in drug resistance exhibited by *H. pylori* in the Nanjing region of China during the preceding five years. Another important objective is to investigate the influence of levofloxacin medication history on genotypic and phenotypic resistance.

**Methods:**

This research screened 4277 individuals diagnosed with *H. pylori* infection between April 2018 and May 2023. The phenotype and genotypic resistance were evaluated using the Kirby-Bauer disk diffusion and PCR method.

**Results:**

The most recent primary resistance rates for metronidazole, clarithromycin, levofloxacin, amoxicillin, furazolidone, and tetracycline were recorded at 77.23% (2385/3088), 37.24% (1150/3088), 27.72% (856/3088), 0.52% (16/3088), 0.19% (6/3088), and 0.06% (2/3088), respectively. For the recent five years, we observed a notable upsurge in the rate of metronidazole resistance and a slight elevation of clarithromycin and levofloxacin resistance. The documented resistance rates to single-drug, dual-drug, triple-drug, and quadruple-drug regimens were 35.39%, 28.32%, 25.72%, and 0.21%, respectively. The prevalence of multidrug-resistant strains escalated, rising from 37.96% in 2018 to 66.22% in 2023. The rate of phenotypic and genotypic resistance rate (57.10% and 65.57%) observed in strains obtained from patients without a levofloxacin treatment history was significantly lower than the rate in strains obtained from those with a history of levofloxacin treatment (88.73% and 94.74%). The prevailing *gyrA* mutations were primarily N87K (52.35%, 345/659), accompanied by D91N (13.96%, 92/659), and closely followed by D87G (10.77%, 71/659). For *gyrA* mutations, the 91-amino acid mutants exhibit a higher likelihood of discrepancies between phenotypic and genotypic resistance than the 87-amino acid mutants.

**Conclusion:**

The extent of antibiotic resistance within *H. pylori* remains substantial within the Nanjing region. If levofloxacin proves ineffective in eradicating *H. pylori* during the initial treatment, its use in subsequent treatments is discouraged. The employment of levofloxacin resistance genotype testing can partially substitute conventional antibiotic sensitivity testing. Notably, predicting phenotypic resistance of levofloxacin through PCR requires more attention to the mutation type of *gyrA* to improve prediction accuracy.

## Introduction

Since its discovery, *Helicobacter pylori* (*H. pylor*i) has emerged as one of the most widespread microorganisms worldwide. The overall prevalence of *H. pylori* worldwide is reported to be 44.3%, with developing countries exhibiting a higher prevalence (50.8%) compared to developed countries (34.7%). (Zamani et al., 2018) In addition to its role in chronic active gastritis and peptic ulcers, *H. pylori* is the most significant identified risk factor for gastric cancer and mucosa-associated lymphoid tissue lymphoma (MALT). Notably, in China, stomach cancer holds the third position for males and the second position for females in terms of malignant tumor occurrences. (Azzaya et al., 2020) Consequently, the infection caused by *H. pylori* has evolved into a substantial public health concern that requires immediate attention. (Shu et al., 2022) The escalation of antimicrobial resistance in recent times underscores the imperative to enhance the efficacy of *H. pylori* treatment, thereby emphasizing the urgency of addressing this pivotal concern (Kuo et al., 2021; Azrad et al., 2022).

The Maastricht VI/Florence consensus report advocates for the incorporation of routine antibiotic sensitivity testing prior to commencing *H. pylori* eradication treatment. This approach is recommended to facilitate the accurate selection of treatment strategies. (Malfertheiner et al., 2022) At present, a limited array of antibiotics, namely amoxicillin, clarithromycin, metronidazole, levofloxacin, tetracycline, and furazolidone, remains effective in combatting *H. pylori.* Nevertheless, the extensive utilization of these constrained antibiotics has hastened the emergence of antibiotic resistance. (Lee et al., 2019; Jiang et al., 2022) China harbors a substantial population afflicted by *H. pylori* infection, revealing a complex landscape of antibiotic resistance (Xie and Lu, 2015).

The eradication regimen incorporating levofloxacin presents a promising approach. It not only stands as a primary therapeutic choice for addressing *H. pylori* infection but also assumes the role of a rescue program when needed. (Miyachi et al., 2006) A meta-analysis showed that 14-day levofloxacin-based sequential therapy was the most efficient therapy globally. (Zamani et al., 2022) However, notable variations exist in the susceptibility of *H. pylori* to levofloxacin across diverse global regions. A study reported that the overall mean prevalence of primary *H pylori* resistance was 18% for levofloxacin in the Asia-Pacific region. (Kuo et al., 2017) But in China, the prevalence of primary resistance to levofloxacin has reached 34.21%. (Zhong et al., 2021) Given the significant prevalence of levofloxacin resistance, it is crucial to address two fundamental aspects: firstly, gaining a comprehensive understanding of the phenotypic resistance variations to levofloxacin in the local region over recent years, and secondly, scrutinizing the patterns of gene mutations associated with levofloxacin resistance.

In light of the escalating drug resistance observed in *H. pylori*, there’s an immediate need to monitor and gather data within the local region consistently. Our center previously reported a cross-sectional study from 2018 to 2021, summarizing the drug resistance of *H. pylori* and genotypic resistance of clarithromycin in Nanjing. (Jiang et al., 2022) However, there is limited available data concerning the prevalence and underlying mechanisms of levofloxacin resistance within the local area. The present study delved into the variations in the percentage of phenotypic resistance exhibited by *H. pylori* towards six commonly administered antibiotics over the past five years. Our emphasis centered on investigating the phenotype resistance profiles specifically related to levofloxacin. Subsequently, we evaluated the influence of the treatment eradication history employing levofloxacin-based regimens and the frequency of eradication attempts on the rate of phenotypic drug resistance.

Our research also focuses on the genotypic resistance of levofloxacin, in addition to phenotypic resistance. The development of drug resistance in *H. pylori* is primarily attributed to mutations that modify crucial drug targets or hinder their effectiveness. Evidence indicates that the resistance of *H. pylori* to fluoroquinolones arises due to specific point mutations occurring in the quinolone resistance determining region (QRDR) of the *gyrA/B* gene. The *gyrA/B* gene encodes the A and B subunits of DNA gyrase. As of now, various mutation patterns within this gene have been documented. (Moore et al., 1995; Dascalu et al., 2023) The predominant mutation types observed in the *gyrA* gene of *H. pylori* in China are Asn87Lys, followed by Asp91Asn and Asp91Gly. (Zhong et al., 2021) The genotypic and phenotypic resistance of levofloxacin exhibit a strong correlation, thereby suggesting the potential for predicting the phenotypic resistance of levofloxacin through the detection of *gyrA* mutations. (Xiong et al., 2023) Enhancing the precision of the above prediction can be achieved by analyzing the various patterns of *gyrA* mutations observed in recent years and specifically investigating the differential effects of mutations at positions 87 and 91 on the phenotypic resistance of levofloxacin. In this study, a comprehensive analysis was undertaken to elucidate the specific mutations with substantial significance in conferring levofloxacin resistance within *H. pylori*.

The present study provides an up-to-date analysis of drug resistance patterns of *H. pylori* in the Nanjing region of China, specifically examining the phenotypic and genotypic resistance to levofloxacin. The findings of this research will provide assistance for the rapid diagnosis and personalized treatment of *H. pylori*.

## Materials and methods

### Study population

**Figure 1:**
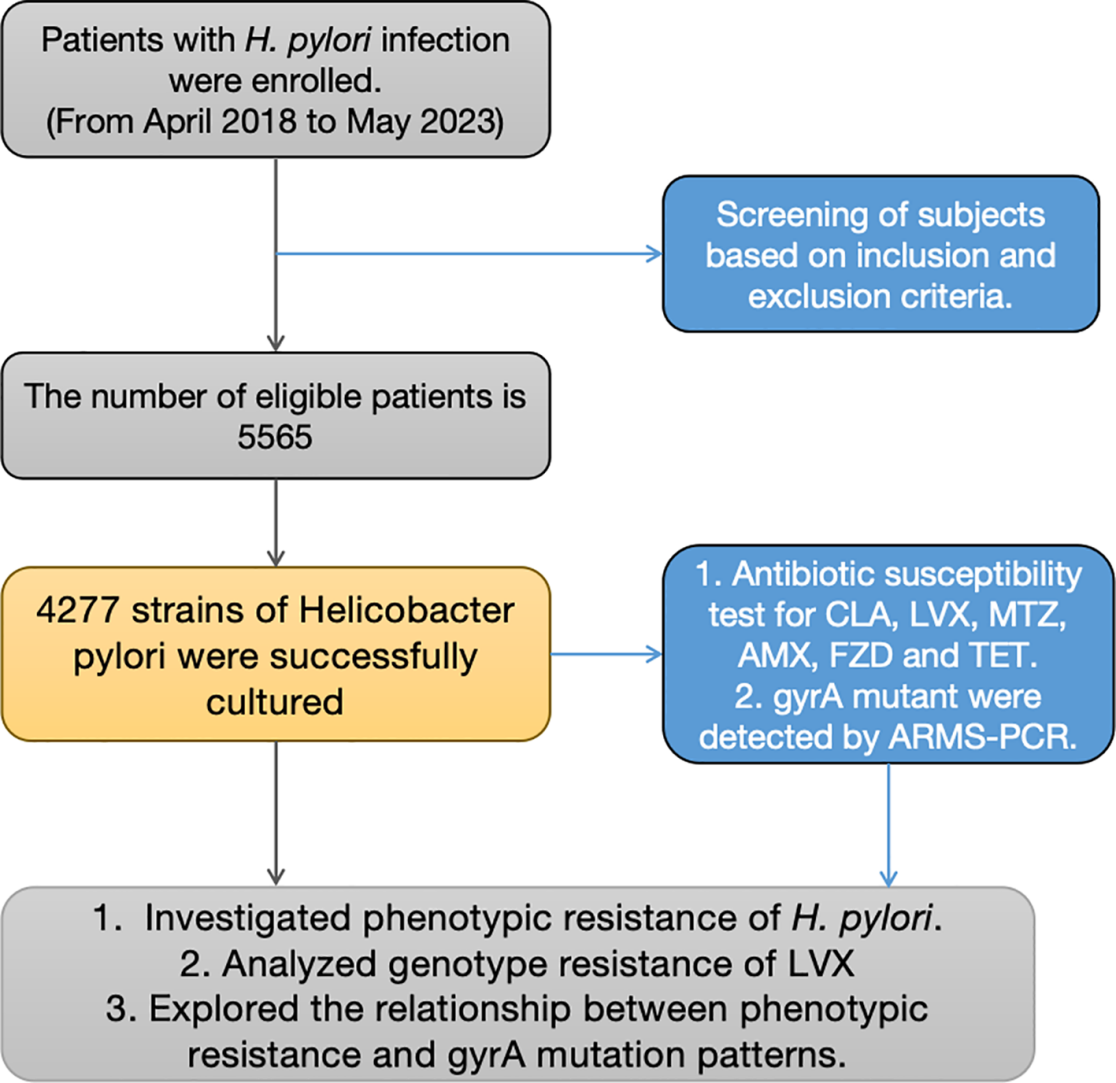
Flowchart depicting the study design. * RUT, rapid urease test; AMX, Amoxicillin; CLA, Clarithromycin; LVX, Levofloxacin; MTZ, Metronidazole; FZD, Furazolidone; TET, tetracycline.

Between April 2018 and May 2023, 5565 individuals who participated in the study underwent upper endoscopy at Nanjing First Hospital. Inclusion criteria: 1) Those individuals participating were required to exhibit at least one positive result from either a 13C-urea breath test, rapid urease test, or pathological examination for *H. pylori;* 2) Participants who provided informed consent were requested to submit gastric mucosa specimens. Those who had consumed antibiotics, proton-pump inhibitors (PPIs), H2 receptor blockers, or bismuth salts within the four weeks preceding the endoscopy were excluded from the study. The successful cultivation of strains was achieved for 4277 participants. (**Figure 1**) Besides, 552 patients were excluded from this study due to antibiotic or proton-pump inhibitor use. The study underwent review and approval by the Ethics Committee at Nanjing First Hospital.

### Sample acquisition

Gastric mucosa samples were collected from patients using a gastroscope. For each patient, two biopsy specimens from the gastric antrum mucosa and one biopsy specimen from the gastric corpus mucosa were placed into a single vial.

### *H. pylori* culture

*H. pylori* was cultured on a Columbia agar plate enriched with 5% sheep blood. The inclusion of vancomycin (1 mg/ml), polymyxin B (0.5 mg/ml), and amphotericin B (0.5 mg/ml) in the agar was essential to inhibit the growth of various bacteria. Culturing bacteria involved placing the plate in an incubator with 10% carbon dioxide and 5% oxygen. This microaerobic environment was maintained at 37°C for 96 to 120 hours. Following cultivation, the obtained isolates or colonies underwent essential procedures such as Gram staining, catalase, and urease testing.

### Phenotypic antibiotic-resistance testing

The Kirby-Bauer disk diffusion method was employed to ascertain the phenotypic resistance of the strains. The antibiotic disks utilized in this study encompassed clarithromycin (15μg), levofloxacin (5μg), amoxicillin (10μg), furazolidone (100μg), tetracycline (30μg), and metronidazole (5μg).

A 0.5 McFarland standard of *H. pylor*i suspension was applied onto a culture plate to initiate the process. After the agar medium was prepared, antibiotic disks were gently pressed onto its surface. The diameter of the resulting inhibitory zone was then measured to determine the level of *H. pylori* resistance to the specific antibiotic: (Megraud et al., 1999; Zhong et al., 2021) *H. pylori* ATCC43504 was employed for quality control purposes. ATCC43504 is a strain endorsed by CLSI M45 as a quality control measure for assessing drug sensitivity in *H. pylori.* CLSI guidelines mandate that researchers perform drug sensitivity testing concurrently on both ATCC43504 and the target bacteria. The reliability of the drug sensitivity test results is contingent upon the normal sensitivity exhibited by ATCC43504, falling within the designated margin of error (CLSI, 2015).

### gyrA mutations of levofloxacin-resistant *H. pylori* isolates

The method for detecting *gyrA* mutations was previously documented by Zhong et al.(Zhong et al., 2021) In our study, the gastric mucosa obtained from the patient’s stomach was directly immersed in the liquid transport medium. DNA was extracted from the specimens in the liquid transport medium using a HiPure bacterial DNA kit (Magen Biotech, Guangzhou, China). The extracted DNA was used as a template for PCR. In levofloxacin-resistant strains, mutations were identified within the quinolone resistance determining region (QRDR) at positions 87 and 91. PCR was performed to amplify the *gyrA* gene, which has been reported to be associated with levofloxacin resistance. The PCR system was comprised of 5ng DNA, 25 μL 2x HiEff PCR Master Mix (with dye), 2 μL forward primers (10 μmol), and 2 μL reverse primers (10 μmol). The amplification procedure involved pre-denaturation at 94°C for 5 minutes, followed by denaturation at 94°C for 10 seconds, annealing at 55°C for 20 seconds, extension at 72°C for 50 seconds and final extension at 72°C for 5 min. This amplification cycle was repeated for a total of 40 cycles. The PCR products were sequenced using Sanger sequencing, as previously described. The forward primer was 5’-GGCGTATTTTGTATGCGATGC-3’ and the reverse primer was 3’-GAAAGTGCGGGCCAAAGTG-5’.

### Statistical analysis

Data analysis was executed using IBM SPSS 26.0 (Chicago, Illinois, USA). Percentages were utilized to portray the resistance of *H. pylori* to antibiotics. The agreement between antibiotic-resistant phenotypes and genotypes was evaluated using the kappa consistency test. In the realm of statistical analysis, the kappa coefficient is bounded within the range of -1 to 1. Generally, a kappa value surpassing 0.75 signifies a notable level of consistency. And the closer the kappa value is to 1, the higher the level of agreement between the above two diagnostic methods for determining levofloxacin resistance.

## Results

### *H. pylori* antibiotic susceptibility

Out of the entire cohort of 5565 subjects assessed between 2018 and 2023, a flourishing culture of *H. pylori* was achieved in 4277 individuals, constituting 74.96% of the total. We compiled the rates of resistance exhibited by *H. pylori* towards six antibiotics in Nanjing. Metronidazole has the highest resistance rate at 80.08% (3425/4277). Simultaneously, the resistance rates observed for levofloxacin and clarithromycin were 37.27% (1594/4277) and 49.78% (2129/4277), respectively. It’s noteworthy to highlight that the frequencies of drug resistance concerning amoxicillin (0.61%, 26/4277), furazolidone (0.23%, 10/4277), and tetracycline (0.05%, 2/4277) remain relatively low.

### Antibiotic resistance changes in *H. pylori*

**Figure 2:**
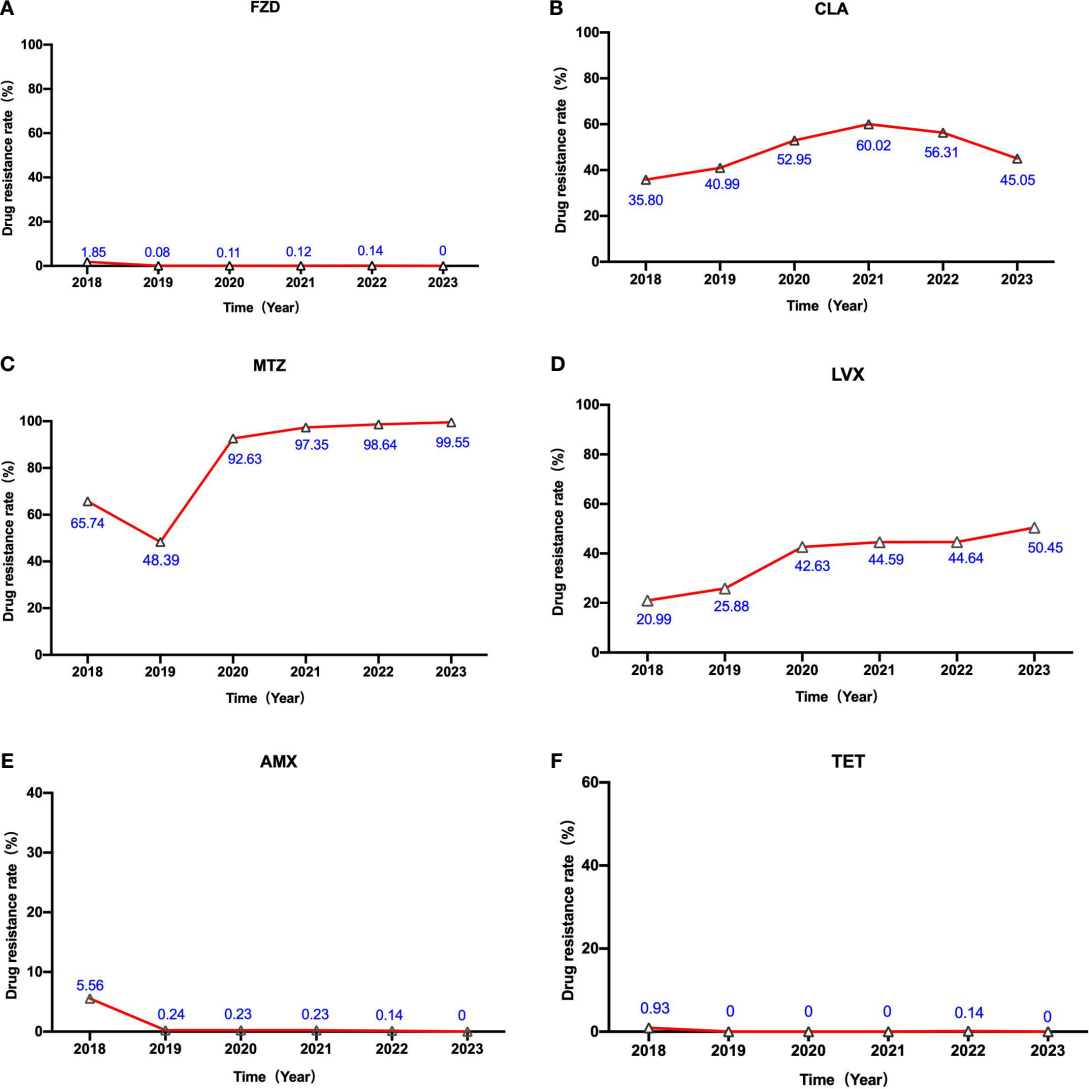
The alterations in *H. pylori’s* susceptibility to six different antibiotics over the course of the previous five years. The labels **(A–F)** represent the alterations in *H. pylori’s* susceptibility to Furazolidone (FZD) , Clarithromycin (CLA) , Metronidazole (MTZ), Levofloxacin (LVX), Amoxicillin(AMX) and Tetracycline (TET) from 2018 to 2023, respectively.

Following that, we analyzed the changes in *H. pylori*’s susceptibility to six distinct antibiotics spanning the last five years. A noticeable escalation in the rate of phenotypic resistance to levofloxacin is evident across this period (as illustrated in **Figure 2**). The resistance rate of levofloxacin underwent a substantial rise, ascending from a relatively modest 20.99% in 2018 to a concerning 50.45% in 2023. In 2021, the resistance rate of clarithromycin peaked at 60.02%, followed by a slight decline in the subsequent two years. Starting in 2019, a notable surge in metronidazole resistance has been observed, with rates approaching nearly 100%. However, it is gratifying to keep that the resistance levels of amoxicillin, furazolidone, and tetracycline have remained consistently low over the past five years, exhibiting no significant fluctuations.

### Effect of previous eradication trials on phenotypic resistance rate

The 4277 participants were classified into three distinct groups. The first group encompassed individuals (n=3088) who had not undergone any eradication treatment previously. The second group consisted of participants (n=417) who had received eradication treatment once only. Lastly, the third group comprised individuals (n=722) who had undergone multiple rounds of eradication treatment.

The resistance of *H. pylori* to clarithromycin, levofloxacin, and metronidazole demonstrated an association with the frequency of prior eradication attempts. Specifically, patients with a history of eradication displayed considerably elevated resistance rates for clarithromycin (93.21%), levofloxacin (77.70%), and metronidazole (96.68%) in comparison to those without any previous eradication attempts. Conversely, the resistance rates of amoxicillin, furazolidone, and tetracycline did not exhibit a pronounced correlation with the eradication history (**Table 1**).

**Table 1:** Antibiotic phenotypic resistance rates of *H. pylori* before treatment or after different times of treatment in Nanjing, China.

Treatment History	Phenotypic resistance rate (%)					
	Clarithromycin	Levofloxacin	Metronidazole	Amoxicillin	Furazolidone	Tetracycline
**No treatment history** **(73.05%, 3088/4227)**	37.24 (1150/3088)	27.72 (856/3088)	77.23 (2385/3088)	0.52 (16/3088)	0.19 (6/3088)	0.06 (2/3088)
**for the second-time treatment** **(9.87%, 417/4227)**	73.38 (306/417)	42.45 (177/417)	82.01 (342/417)	0.72 (3/417)	0.48 (2/417)	0.24 (1/417)
**for treatment more than 2 times** **(17.08%, 722/4227)**	93.21 (673/722)	77.70 (561/722)	96.68 (698/722)	0.97 (7/722)	0.28 (2/722)	0.14 (1/722)

### Drug resistance pattern of *H. pylori* in Nanjing

**Figure 3:**
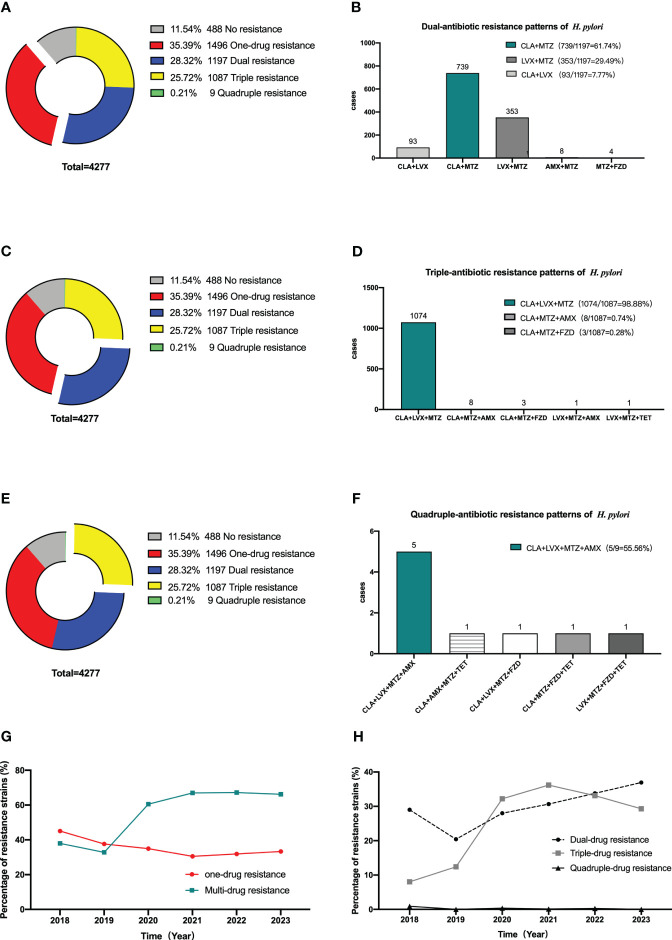
Multi-antibiotics resistance patterns of *H pylori.* **(A, B)** Dual-antibiotics resistance patterns. **(A)** Composition of antibiotics resistance patterns. The blue part represented the proportion of total dual-antibiotics resistance patterns, accounting for 28.32% (1197/4227). **(B)** Composition of dual antibiotics resistance patterns. **(C, D)** Triple-antibiotics resistance patterns. **(C)** Composition of antibiotics resistance patterns. The yellow part represented the proportion of total triple-antibiotics resistance patterns, accounting for 25.72% (1087/4227). **(D)** Composition of triple-antibiotics resistance patterns. **(E, F)** Quadruple-antibiotics resistance patterns. **(E)** Composition of antibiotics resistance patterns. The purplish red part represented the proportion of total quadruple-antibiotics resistance patterns, accounting for 0.21% (9/4227). **(F)** Composition of quadruple-antibiotics resistance patterns. AMX, amoxicillin; CLA, clarithromycin; FZD, furazolidone; MTZ, metronidazole; LVX, levofloxacin; TET, tetracycline. **(G)** Alterations in the proportion of one-drug and multi-drug resistant *H. pylori* strains in the past 5 years. **(H)** Alterations in the proportion of dual-drug, triple-drug, and quadruple-drug resistant *H. pylori* strains in the past 5 years.

Among the 4277 strains scrutinized, 488 (11.54%) were entirely susceptible, whereas 1496 (35.39%) evidenced resistance to a sole drug. Moreover, 1197 strains (28.32%) displayed resistance to two drugs, 1087 strains (25.72%) indicated opposition to three drugs, and a mere nine strains (0.21%) were identified as resistant to four drugs. Within the subset of dual-resistant strains, the predominant combinations were clarithromycin+metronidazole (61.74%), followed by levofloxacin metronidazole (31.78%), and clarithromycin+levofloxacin (29.49%). Over the course of the previous five years, there has been a decline in the prevalence of single drug resistant strains, juxtaposed with a persistent rise in the prevalence of multi drug resistant strains. There was a notable rise in the proportion of double resistant strains, whereas the proportion of triple and quadruple resistant strains experienced a marginal change (**Figure 3**).

### Analysis of *gyrA* resistance mutations

The current study encompassed the analysis of *gyrA* mutations in a cohort of 1076 individuals from 2018 to 2023. Within this sample, the prevalence of individuals carrying *gyrA* mutations was 61.25% (659/1076). Notably, patients without eradication therapy (n=312) exhibited a *gyrA* mutation prevalence of 37.18%. In comparison, those who had received eradication therapy once (n=166) had a higher incidence of 47.59%. Remarkably, patients who had undergone eradication treatments more than twice (n=598) displayed a significantly elevated occurrence of *gyrA* mutations, reaching 77.59%.

In our study, the total number of patients who have treatment-experienced is 1139. However, only 776 among them registered their previous therapeutic medication. These 776 patients were categorized into two groups based on their history of levofloxacin usage. Among these groups, 142 individuals had previously undergone levofloxacin eradication therapies, while 634 patients had not been subjected to any levofloxacin-based eradication treatment. Simultaneously, we attempted to analyze the potential correlation between a prior administration of levofloxacin and the occurrence of *gyrA* mutations. Although the present investigation involved the examination of *gyrA* mutations in a cohort of 1076 individuals, only 509 patients registered their previous therapeutic medication. The 509 participants were classified into two cohorts according to their prior utilization of levofloxacin. Out of the two aforementioned groups, a total of 114 individuals had previously undergone levofloxacin eradication therapies, whereas 395 patients had not received any levofloxacin-based eradication treatment. The research findings revealed that the proportion of strains exhibiting *gyrA* mutations among patients without a history of levofloxacin treatment was significantly lower (65.57%) compared to strains isolated from individuals who had undergone levofloxacin treatment (94.74%). Furthermore, the rate of phenotypic resistance (57.10%) observed in strains obtained from patients without a levofloxacin treatment history was significantly lower than the rate in strains obtained from those with a history of levofloxacin treatment (88.73%) (**Table 2**).

**Table 2:** Analysis of the phenotype and genotypic resistance rate of *H. pylori* isolated from patients with and without levofloxacin treatment history.

	Genotypic resistance to Levofloxacin(%)		Phenotypic resistance to Levofloxacin(%)	
	Without *gyrA* mutation	With *gyrA* mutation	Sensitive	Resistance
**No history of Levofloxacin eradication**	34.43(136/395)	65.57(259/395)	42.90(272/634)	57.10(362/634)
**Levofloxacin eradication history**	5.26(6/114)	94.74(108/114)	11.27(16/142)	88.73(126/142)

Subsequently, we analyzed the *gyrA* mutation patterns within 659 *H. pylori* isolates. Throughout this investigation, a total of 20 distinct *gyrA* mutations were identified. The most frequently observed mutation was N87K (52.35%, 345/659), trailed by D91N (13.96%, 92/659) and D91G (10.77%, 71/659). The N87K mutation involves an amino acid alteration at position 87, converting Asn to Lys due to a T to A or G substitution at codon 87 (AAT).

Among these 20 mutation types, a total of 7 were single-site mutations (91.81%, 605/659), 12 were double-site mutations (8.04%, 53/659), and there was only one instance of a triple-site mutation (0.15%, 1/659). The complete spectrum of *gyrA* mutation types is presented in **Table 3**.

**Table 3:** Cases of different mutation types in *gyrA* of *H. pylori*.

Frequency of *gyrA* mutation (%) (n=659)		*gyrA* mutations data		
		*gyrA* mutation and position	Amino acid change	Nucleotide change
1	345 (52.35)	N87K	Asn→Lys	AAT→AA **A/**AA **G**
2	92 (13.96)	D91N	Asp→Asn	GAT→ **A** AT
3	71 (10.77)	D91G	Asp→Gly	GAT→G **G** T
4	46 (6.98)	N87I	Asn→Ile	AAT→A **TC**/A **T** T
5	40 (6.07)	N91Y	Asp→Tyr	GAT→ **T** AT/**A** AT
6	9 (1.37)	D87Y	Asn→Tyr	AAT→ **T** A **C**/**T** AT
7	2 (0.30)	N87R	Asn→Arg	AAT→A **GG**/**CG** T
8	14 (2.12)	N87K, D91G	Asn→Lys, Asp→Gly	AAT→AA **A/**AA **G**, GAT→G **G** T
9	9 (1.37)	N87K, D91N	Asn→Lys, Asp→Asn	AAT→AA **A/**AA **G**, GAT→ **T** AT
10	6 (0.91)	N87K, D91Y	Asn→Lys, Asp→Tyr	AAT→AA **A/**AA **G**, GAT→ **T** AT/**A** AT
11	5 (0.76)	D91N, D91G	Asp→Asn, Asp→Gly	GAT→ **A** AT, GAT→G **G** T
12	4 (0.61)	N91Y, D91G	Asp→Tyr, Asp→Gly	GAT→ **T** AT, GAT→G **G** T
13	4 (0.61)	N87I, N87K	Asn→Ile, Asn→Lys	AAT→A **TC**/A **T** T/ATA, AAT→AA **A/**AA **G**
14	3 (0.46)	N91Y, D91N	Asp→Tyr, Asp→Asn	GAT→ **T** AT, GAT→ **A** AT
15	3 (0.46)	N87I, D91N	Asn→Ile, Asp→Asn	AAT→A **T** T, GAT→ **A** AT
16	2 (0.30)	N87I, D91Y	Asn→Ile, Asp→Tyr	AAT→A **TC**/A **T** T, GAT→ **A** AT
17	1 (0.15)	N87I, D91G	Asn→Ile, Asp→Gly	AAT→A **TC**, GAT→G **G** T
18	1 (0.15)	D87Y, D91N	Asn→Tyr, Asp→Asn	AAT→ **T** AT, GAT→ **A** AT
19	1 (0.15)	N87K, D87Y	Asn→Lys, Asn→Tyr	AAT→AA **A/T** A **C**
20	1 (0.15)	D91G, D91N, N91Y	Asp→Gly, Asp→Asn, Asp→Tyr	GAT→G **G** T, GAT→ **A** AT, GAT→ **T** AT

The highlighted values represent the nucleotide mutations.

In the context of N87K/I/R and D87Y mutant strains, the proportion of susceptible strains stands at 1.77%, whereas for D91N/G and N91Y mutant strains, the percentage of susceptible strains rises to 8.37%. This observation suggests that mutants at codon 91 may be more prone to inconsistent of phenotypic and genotypic resistance than mutants at codon 87.

**Figure 4:**
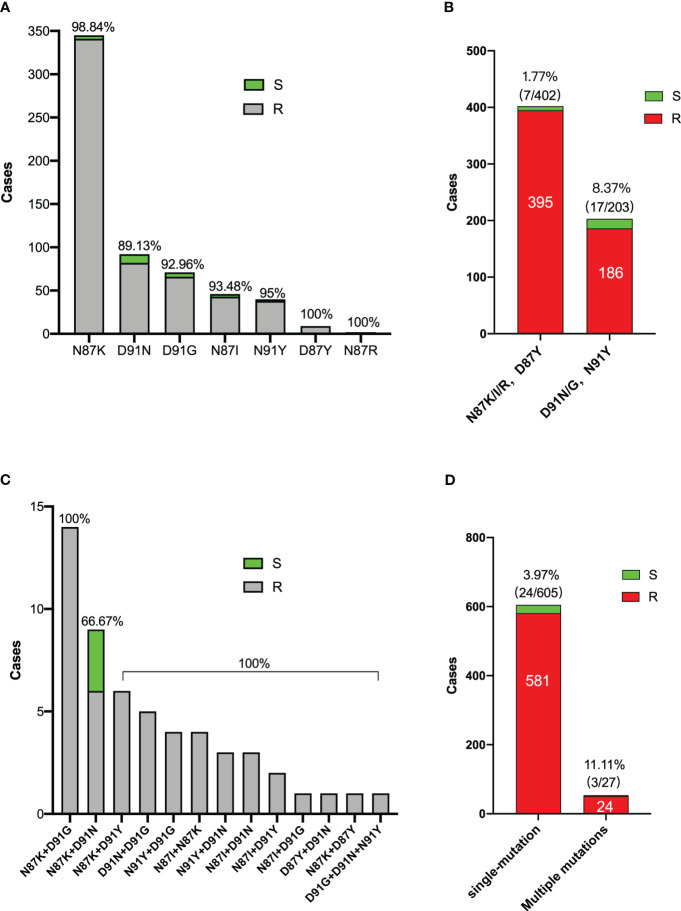
Characters of *gyrA* mutation patterns of 659 *H. pylori* strains. **(A)** Distribution of levofloxacin sensitive and resistant bacteria in single mutation strains. **(B)** Levofloxacin sensitive bacteria proportions between mutants in codon 87 and codon 91. **(C)** Distribution of levofloxacin sensitive and resistant bacteria in multiple mutation strains. **(D)** Levofloxacin sensitive bacteria proportions between single and multiple mutants. S: Levofloxacin-sensitive strains; R: Levofloxacin-resistance strains.

Within the N87K+D91N mutant, three levofloxacin-sensitive strains (33.33%, 3/9) were identified, whereas no phenotypically sensitive strains were detected among all other double and triple mutants. Moreover, multiple mutant strains did not exhibit a greater prevalence of phenotypic resistance than single mutant strains (**Figure 4**).

### The consistency of antibiotic sensitivity test results and PCR results

Levofloxacin resistance has reached a relatively notable level in Nanjing. In this clinical context, PCR represents a straightforward and effective method for detecting *gyrA* mutations, enabling a more precise selection of antibiotics. Compared to the established gold standard drug sensitivity test, PCR demonstrates a sensitivity of 96.78% and a specificity of 93.62%.

Moreover, a significant and strong correlation was observed between the results obtained from PCR and the outcomes of drug sensitivity tests. The correlation coefficient (κ) was determined to be 0.910, accompanied by a highly significant p-value of less than 0.001, as indicated in **Table 4**.

**Table 4:** Comparison of the antibiotic susceptibility test and PCR detection of *H. pylori* antibiotic resistance gene mutation.

Antibiotic susceptibility test (gold standard)		PCR test result			Kappa value	Asymp. Std. Error	Approx. Tb	Approx. Sig.
		Mutation	Wild	Total				
**Levofloxacin**	**R**	632	21	653	0.910	0.013	30.215	<0.001
	**S**	27	396	423				
	**Total**	659	417	1076				

## Discussion

As a notable contributor to the pathogenesis of upper gastrointestinal diseases, *H. pylori* assumes a critical role. The prevalence of *H. pylori* infection ranges from 25% to 50% in developed countries and can reach as high as 80% in developing countries. (Jiang et al., 2022) Particularly in mainland China, the overall occurrence rate of *H. pylori* is reported at 44.2%, affecting approximately 589 million individuals (Ren et al., 2022).

The escalating antibiotic resistance exhibited by *H. pylori* has elicited global concern. China, too, confronts a significant challenge of antibiotic resistance, which presents grave implications for the effective treatment of *H. pylori* infections. (Zhong et al., 2021; Xu et al., 2022) Despite recent studies suggesting that high-dose dual therapy (HDDT) demonstrates similar effectiveness and adherence compared to bismuth-containing quadruple therapy (BQT) or standard triple treatment, BQT remains the predominant treatment approach for *H. pylori* infection in China (Chey et al., 2022; Zou et al., 2022).

Nanjing, situated in eastern China, is a metropolis with a populace exceeding 10 million. The prevalence of *H. pylori* infection in this region is relatively high. The imperative need arises to monitor *H. pylori* drug resistance within the region continuously. Such monitoring is a vital resource for local healthcare professionals, aiding them in making well-informed antibiotic selections for effective treatments.

Based on these considerations, our center assessed the alterations in *H. pylori* isolate resistance to frequently employed antibiotics spanning 2018 to 2023. Considering that the levofloxacin based BQT is one of the predominant treatment strategies for eradicating *H. pylori*, it is crucial to promptly obtain current data on antibiotic resistance within the specific region, with a specific focus on the resistance rate of levofloxacin against *H. pylori*. Besides, many studies have already confirmed that the genotypic and phenotypic resistance of levofloxacin exhibit a strong correlation. The objective of this study is to enhance the precision of prognosticating phenotypic resistance to levofloxacin through *gyrA* mutations by examining diverse patterns of *gyrA* mutations observed in recent years and investigating the distinct impacts of mutations at positions 87 and 91 on phenotypic resistance to levofloxacin.

A previous study conducted by our center uncovered that within the timeframe spanning 2018 to 2021, the pre-treatment resistance rates for metronidazole, clarithromycin, levofloxacin, amoxicillin, furazolidone, and tetracycline in Nanjing were documented at 67.19%, 35.99%, 24.23%, 0.76%, 0.28%, and 0.09%, respectively. (Zhong et al., 2021) In our study, the most recent primary resistance rates for the aforementioned antibiotics were recorded at 77.23%, 37.24%, 27.72%, 0.52%, 0.19%, and 0.06%, respectively. Our research findings indicate a notable upsurge in the rate of metronidazole resistance in comparison to previously documented data. Additionally, a slight elevation was observed in the resistance rates for clarithromycin and levofloxacin, whereas a decline was noted in the rates of resistance to amoxicillin, furazolidone, and tetracycline. It is noteworthy to observe that the prevalence of multidrug-resistant *H. pylori* is progressively escalating over time, potentially attributable to the utilization of non-standard treatment approaches and the excessive use of antibiotics. For patients who experienced initial treatment failure, the resistance rates for clarithromycin, levofloxacin, and metronidazole significantly increased. Furthermore, it was revealed that an escalation in the number of eradication attempts was correlated with a corresponding rise in drug resistance rates. This exacerbates the already complex issue of multiple antibiotic resistance.

In the intricate landscape of drug resistance, we advocate the idea that all individuals diagnosed with *H. pylori* infection should ideally undergo antibiotic sensitivity testing to enable the formulation of targeted treatment plans. Nevertheless, implementing such an approach may not be feasible for all patients. For those initiating their treatment journey, a regimen incorporating levofloxacin or clarithromycin for eradication is a favorable choice in situations where sensitivity testing cannot occur. Because in many local secondary and community hospitals, levofloxacin and clarithromycin are commonly used due to their accessibility.

It has been documented that in China, the primary and secondary antibiotic resistance rates for levofloxacin in *H. pylori* were 34.21% and 61.58%, respectively. (Zhong et al., 2021) In contrast, the resistance rate of levofloxacin in Nanjing is higher. Our study has demonstrated a pronounced correlation between the history of eradication attempts using levofloxacin-containing regimens, the escalation of phenotypic drug resistance rates, and the prevalence of *gyrA* gene mutations. Consequently, if levofloxacin proves ineffective in eradicating *H. pylori* during the initial treatment, it is advisable to avoid its usage in subsequent treatment strategies. In light of the prevailing drug resistance observed in *H. pylori*, we recommend that local healthcare practitioners possess a comprehensive understanding of the specific drug resistance landscape within their region. Physicians should diligently inquire about patients’ medication history before making prescription decisions, allowing for the formulation of personalized treatment strategies that align with the individual’s unique circumstances.

Furthermore, the examination of levofloxacin’s genotypic resistance is also a central aspect of the present study’s discourse. Firstly, PCR exhibited a substantial correlation with levofloxacin susceptibility tests, indicating its potential utility in clinical settings for guiding the appropriate usage of levofloxacin. This outcome aligns with prior research findings, reinforcing the validity and applicability of PCR as a reliable method for guiding treatment decisions related to levofloxacin. (Trespalacios-Rangel et al., 2016; Hu et al., 2023) Consistent with previous studies conducted in our nation, (Zhong et al., 2021) this investigation primarily focused on identifying point mutations occurring at amino acids 87 (resulting in substitutions from Asn to Lys, Ile, Try, or Arg) and 91 (resulting in substitutions from Asp to Asn, Gly, or Tyr) within the strains under examination. However, distinct from earlier studies, our research documented an added dimension by identifying 53 cases of *gyrA* double mutations and one instance of triple mutation. Furthermore, our findings revealed that the occurrence of mutations in codon 87 surpassed that in codon 91. Again, among the sensitive strains, 1.77% displayed a point mutation in codon 87, while a more significant proportion of 8.37% exhibited sensitivity despite a point mutation in codon 91.

Except for the N87K+D91N mutation, all isolates carrying multiple *gyrA* mutations resisted levofloxacin. Most instances showing discrepancies between antibiotic sensitivity tests and *gyrA* mutation detection outcomes were observed in strains featuring point mutations in codon 91. Among these, the most pronounced difference arose in strains with the D91N mutation. An *in vitro* investigation suggested that mutations in codon 87 are more impactful than those in codon 91, and the effects of combined mutations are even more pronounced than single mutations. Moreover, the study found that mutations in codon 91 were reversible, (Wang et al., 2020) indicating potential instability. This inherent instability in codon 91 mutations might explain the variance between antibiotic sensitivity and *gyrA* mutation test outcomes.

Currently, various kits are in development for *gyrA* genetic testing, aiming to predict *H. pylori* resistance to levofloxacin. These kits predominantly target the identification of *gyrA* genes within feces, gastric mucosa, or even gastric juice samples. (Ierardi et al., 2017; Tsuda et al., 2022) Analyzing the specific types of *gyrA* mutations holds the potential to enhance the precision and effectiveness of PCR detection tools. Our study proposes that *gyrA* dual and triple mutations exhibit a significant level of precision in predicting the resistance phenotype of levofloxacin, except for N87K+D91N mutations. This information can contribute to refining PCR detection components, improving sensitivity and specificity within *gyrA* detection kits. The findings of our study have the potential to enhance the precision of clinical practitioners in forecasting levofloxacin phenotypic resistance through gene testing outcomes, consequently providing improved guidance for clinical medication.

Furthermore, a subset of levofloxacin-sensitive strains, totaling 6.38% (27/423), exhibited the presence of *gyrA* mutations at either Asn-87 or Asp-91. On the contrary, 3.22% (21/653) showed an absence of *gyrA* mutation among the levofloxacin-resistant strains. These observed phenomena likely stem from the coexistence of both mutant and wild-type strains. It’s well-established that a single patient can host diverse strains of *H. pylori*. (Kim et al., 2003) Hetero-resistance or genetic heterogeneity may manifest within a patient’s pair of biopsy samples. Furthermore, it is conceivable that a single sample could potentially encompass a mixture of two distinct phenotypic or genotypic populations. This is also the reason why double point mutation was found at the same locus in our study. According to a study conducted by Emmanuelle Cambau, a single biopsy specimen exhibited the presence of a double mutation at codon 91 (D91N+D91G or D91N+D91Y). (Cambau et al., 2009) Further, some reports suggest that N87 wild-type and N87K mutant-type may exist in the same biopsy sample. (Cambau et al., 2009; Binmaeil et al., 2021) The presence of a combination of susceptible and resistant genotypes has been associated with clinical failure. While infections with more than one strain may be associated with more severe disease (Sheu et al., 2009).

This study is not without its limitations. Firstly, the samples were sourced solely from a single center in Nanjing, which may introduce inherent biases to the results. Secondly, the conclusions drawn may not fully encapsulate the broader antibiotic resistance patterns of *H. pylori* across various anatomical locations within the stomach. Thirdly, the study did not determine minimum inhibitory concentrations (MICs) for *H. pylori* isolates, consequently inhibiting the establishment of a definitive link between the *gyrA* mutation patterns and levofloxacin MIC values.

In conclusion, the resistance of *H. pylori* in Nanjing has not improved compared to recent years, with a slight elevation observed in primary resistance to levofloxacin, clarithromycin, and metronidazole. The prevalence of multidrug-resistant strains is progressively escalating. The necessity for antibiotic sensitivity testing before employing these three antibiotics in eradication treatment is highlighted. It is not recommended to reuse the eradication plan containing levofloxacin on the same patient. For evaluating levofloxacin susceptibility in *H. pylori*, a DNA-based screening approach is a promising substitute for conventional culture methods. However, predicting phenotypic resistance of levofloxacin through PCR require more attention to the mutation type of *gyrA* to improve prediction accuracy. Vigilant levofloxacin resistance monitoring remains crucial to formulating effective strategies for eradicating *H. pylori*.

## Data availability statement

The original contributions presented in the study are included in the article/**Supplementary Material**, further inquiries can be directed to the corresponding authors.

## Ethics statement

The studies involving humans were approved by Ethics Committee of Nanjing First Hospital. The studies were conducted in accordance with the local legislation and institutional requirements. The participants provided their written informed consent to participate in this study.

## Author contributions

WW: Data curation, Investigation, Writing – original draft, Writing – review & editing. ZW: Data curation, Investigation, Methodology, Writing – original draft. CL: Data curation, Investigation, Writing – review & editing. ZJ: Formal Analysis, Investigation, Writing – review & editing. ZZ: Conceptualization, Project administration, Supervision, Writing – review & editing. SW: Conceptualization, Project administration, Supervision, Writing – review & editing.
