# Supplementary material for: Antibiotic resistance of Helicobacter pylori in Nanjing, China: a cross-section study from 2018 to 2023
Source: Front Cell Infect Microbiol. 2023 Nov 28;13:1294379. doi: 10.3389/fcimb.2023.1294379 (PMC10714007; doi:10.3389/fcimb.2023.1294379)
Supplement: Supplementary file 1 [file Table_1.docx]

|  | ***gyrA* mutation and position** | **Aminoacid change** | **Nucleotide change patterns** | |
| --- | --- | --- | --- | --- |
|  |  |  | **Most possible mutation sites** | **Other mutation types** |
| 1 | D91N，D91G | Asp→Asn, Asp→Gly | GAT→**A**AT, GAT→G**G**T | GAT-**AG**T（no report），GAT（wild type） |
| 2 | D91N，D91G |  | GAT→**A**AT, GAT→G**G**T | GAT-**AG**T（no report），GAT（wild type） |
| 3 | D91N，D91G |  | GAT→**A**AT, GAT→G**G**T | GAT-**AG**T（no report），GAT（wild type） |
| 4 | D91N，D91G |  | GAT→**A**AT, GAT→G**G**T | GAT-**AG**T（no report），GAT（wild type） |
| 5 | D91N，D91G |  | GAT-**A**AT，GAT-**T**AT | —— |
| 6 | N91Y，D91G | Asp→Tyr, Asp→Gly | GAT→**T**AT, GAT→G**G**T | GAT-**TG**T（no report），GAT（wild type） |
| 7 | N91Y，D91G |  | GAT→**T**AT, GAT→G**G**T | GAT-**TG**T（no report），GAT（wild type） |
| 8 | N91Y，D91G |  | GAT→**T**AT, GAT→G**G**T | GAT-**TG**T（no report），GAT（wild type） |
| 9 | N91Y，D91G |  | GAT→**T**AT, GAT→G**G**T | GAT-**TG**T（no report），GAT（wild type） |
| 10 | N87I，N87K | Asn→Ile, Asn→Lys | AAT→A**TC**, AAT→AA**A** | AAT-A**TA**，AAT-AA**C** |
| 11 | N87I，N87K |  | AAT-AA**G**，AAT-A**T**T | AAT-A**TG**（no report），AAT（wild type） |
| 12 | N87I，N87K |  | AAT→A**TC**, AAT→AA**A** | AAT-A**TA**，AAT-AA**C** |
| 13 | N87I，N87K |  | AAT→A**TC**, AAT→AA**A** | AAT-A**TA**，AAT-AA**C** |
| 14 | N91Y，D91N | Asp→Tyr, Asp→Asn | GAT→**T**AT, GAT→**A**AT | —— |
| 15 | N91Y，D91N |  | GAT→**T**AT, GAT→**A**AT |  |
| 16 | N91Y，D91N |  | GAT→**T**AT, GAT→**A**AT |  |
| 17 | N87K，D87Y | Asn→Lys, Asn→Tyr | AAT→AA**A，**AAT-**T**A**C** | AAT-AA**C**，AAT-**T**A**A**（no report） |
| 18 | D91G，D91N，N91Y | Asp→Gly, Asp→Asn, Asp→Tyr | GAT→G**G**T, GAT→**A**AT, GAT→**T**AT | GAT-**TG**T（no report），GAT-G**G**T，GAT-**A**AT |
|  |  |  |  | GAT-**TG**T（no report），GAT-**AG**T，GAT（wild type） |
|  |  |  |  | GAT-**T**AT，GAT-**AG**T（no report），GAT（wild type） |

**Supplementary Table 1. Analysis of double or triple mutations at the same locus*.***
